# Decoding the Morphological Differences between Himalayan Glacial and Fluvial Landscapes Using Multifractal Analysis

**DOI:** 10.1038/s41598-017-11669-0

**Published:** 2017-09-08

**Authors:** Srimonti Dutta

**Affiliations:** Department of Physics, Behala College, Parnasree Pally, Kolkata, 700060 India

## Abstract

Himalayas is the home to nearly 10,000 glaciers which are mostly located at high and inaccessible region. Digital Elevation Model (DEM) can be effective in the study of these glaciers. This paper aims at providing an automated distinction of glacial and fluvial morphologies using multifractal technique. We have studied the variation of elevation profile of Glacial and Fluvial landscapes using Multifractal Detrended Fluctuation Analysis (MFDFA). Glacial landscapes reveal more complex structure compared to the fluvial landscapes as indicated by fractal parameters degree of multifractality, asymmetry index.

## Introduction

The study of morphological distinction between glacial and fluvial landscapes has been a topic of interest for many years. Various investigations have been undertaken to characterize them^1–7^. Apart from qualitative descriptions, there have been some recent approaches for quantitative description of glacial and fluvial landscapes^8, 9^. Li *et al*.^8^ have shown that the morphology of glacial valley cross-sections can be quantitatively described by power law or quadratic equations. Prasicek *et al*.^9^ have observed that automated characterization of glacial landscapes is possible using multi-scale curvature technique. Brocklehurst^10^ recommended that Digital Elevation Model (DEM) analysis can be effective in distinguishing glacial and fluvial landscapes.

The study of fractal properties of coastlines, river basins, morpho-tectonic features, surface properties of glaciers, etc. can be effective in the identification of various morphological characteristics of landscapes^11–15^. Differences in fractal characteristics of topography can be associated with transitions in dominance of different geo-morphological processes^16^. In this paper we have studied the multifractal properties of the elevation profile of Himalayan glacial and fluvial landscapes using Multifractal Detrended Fluctuation Analysis (MFDFA)^17^.

Though there have been numerous monofractal approaches to the study of the fractal properties of geomorphologies, recent studies have shown that a multifractal approach is more relevant as fractal parameters may vary depending on the locations^18–24^. MFDFA is considered to be an important tool for extracting the multifractal properties of fluctuation pattern and has been successfully applied to diverse fields such as heart rate dynamics, human gait, earthquake signals, and economic time series. Though designed for studying the fluctuation of time series, the technique has been successfully applied in studying multifractality of spatial patterns as well^25–28^. We have chosen the glacial valley around Bara Shigri Glacier as the glacial landscape and the Beas Basin around Manali as the fluvial landscape. Himalayan mountains houses about 10,000 glaciers^29^ located at high and inaccessible region. So Digital Elevation Model (DEM) analysis can be valuable in studying those glaciers. There has been considerable amount of data on Himalayan glaciers^30–34^ near the region of our interest, but the multifractal properties of Himalayan glaciers have not been investigated in past.

We have observed that the glacial landscapes have a more complex structure compared to the fluvial landscapes.

## Description of Data

**Figure 1:**
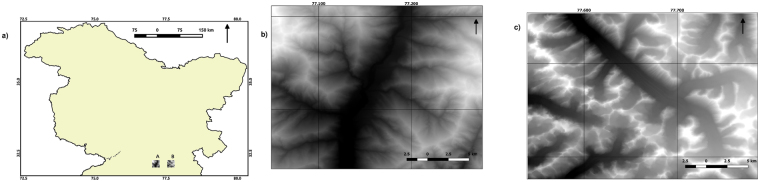
(**a**) Location Map of Selected Regions (**b**) Map of Region A (**c**) Map of Region B. The maps were created by open-source QGIS 2.80 (www.qgis.org). The data used is Shuttle Radar Topography Mission (SRTM) 1 Arc-Second Global digital elevation model (https://lta.cr.usgs.gov/SRTM1Arc).

We analyze the topographic data from two distinct regions of the Western Himalaya where (A) Fluvial and (B) Glacial processes controls the landscape evolution (see location map, Fig. 1(a)). The source of the topographic data is a 1 arc second Digital Elevation Model (DEM) derived from shuttle radar topographic mission (SRTM1)^35^. Each of the 30 km × 30 km squares were extracted from the SRTM1 DEM using QGIS (refer to Fig. 1(b) and (c)). The glacial valleys in B mostly belong to Chandra basin, Lahaul Himalaya, and include a large glacier, namely, Bara Shigri glacier. The region A is located in the upper Beas catchment, around the town of Manali.

**Figure 2:**
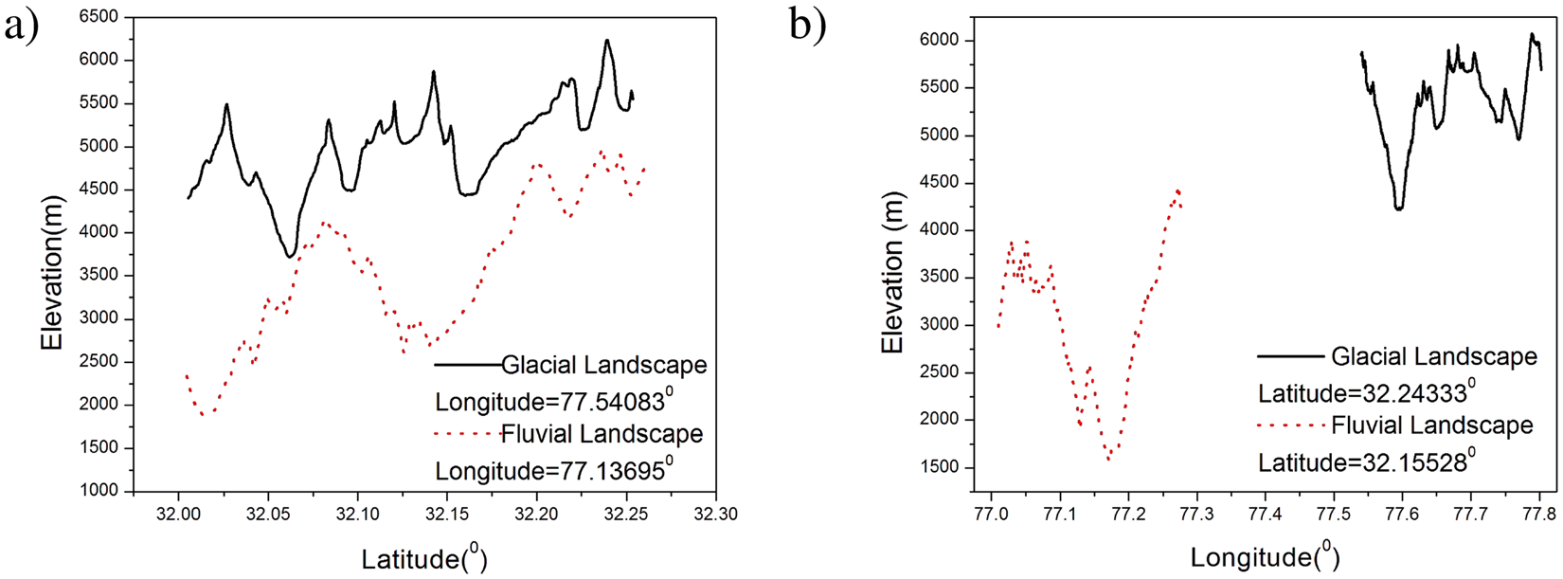
Elevation Profile for Glacial and Fluvial Landscapes. Variation of Elevation with (**a**) Latitude for given Longitude (**b**) Longitude for given Latitude for Glacial and Fluvial Landscape.

Using SRTM1 the latitude(y), longitude(x) and elevation (z) at each point of glacial and fluvial landscapes were extracted. The data was then divided into subsets: variation of elevation with latitude for a fixed longitude (see Fig. 2(a)) and variation of elevation with longitude for fixed latitude (see Fig. 2(b)). Thus we get elevation profiles for all latitudes and longitudes in the given region. Multifractal detrended fluctuation analysis was applied on each set to reveal the multifractal properties of the elevation profile.

## Method of Analysis

The Multifractal Detrended Fluctuation Analysis (MFDFA) methodology, conceived by Kantelhardt *et al*.^17^, was employed to study the fluctuation of the elevation profile of fluvial and glacial landscapes. MFDFA is a generalization of the DFA (Detrended Fluctuation Analysis) methodology introduced by Peng *et al*.^36^. The multifractal formalism of DFA was introduced to overcome the limitations of DFA. It was observed that many records did not reveal simple monofractal scaling which could be described by a single exponent. There existed cross-over scales^37, 38^ in some cases while in other cases scaling behaviour was far more complicated requiring different exponents for different parts of the series^39^. Such different scaling behaviour can also be observed for many interwoven fractal subsets, hence a multitude of scaling exponents is required for a full description of the scaling behaviour, and therefore a multifractal analysis is required. The steps for MFDFA are as follows:

**Step1:** Computing the average

Consider a series given by $x(i)$ for i = 1 ……. *N*, be a non-stationary series of length *N*. Mean is defined as

1 $${x}_{ave}=\frac{1}{N}\sum _{i=1}^{N}x(i)$$

**Step 2:** Computing the integrated series

2 $$Y(i)\equiv \sum _{k=1}^{i}[x(k)-{x}_{ave}]\,\,{\rm{for}}\,\,{\rm{i}}=1\ldots \ldots .N$$

**Step 3:** The integrated series is partitioned to *N*_*S*_ non-overlapping bins (where ${N}_{s}=\,{\rm{int}}(N/s)$, *s* is the length of the bin). Since *N* is not a multiple of *s*, a part of series at the end is left. In order to include this part of the series the entire process is repeated starting from the opposite end. Thus 2*Ns* bins are obtained and for each bin least square fit of the series are performed and variance is determined.

${F}^{2}(s,\nu )=\frac{1}{s}\sum _{i=1}^{s}\{Y[(\nu -1)s+i]-{y}_{\nu }(i){\}}^{2}$ for each bin $\nu ,\nu =1,\ldots .{N}_{s}$, and

${F}^{2}(s,\nu )=\frac{1}{s}\sum _{i=1}^{s}\{Y[N-(\nu -{N}_{s})s+i]-{y}_{\nu }(i){\}}^{2}$ for $\nu ={N}_{s}+1,\ldots \mathrm{..},2{N}_{s}$ where ${y}_{\nu }(i)$ is the least square fitted value in the bin $\nu$. In this case we have used a linear detrending and hence performed a linear least square fit.

**Step4:** Computing fluctuation function

The ${q}^{th}$ order fluctuation function ${F}_{q}(s)$ is obtained after averaging over 2*N*_*s*_ bins.

3 $${F}_{q}(s)=\{\frac{1}{2{N}_{s}}{\sum _{\nu =1}^{2{N}_{s}}[{F}^{2}(s,\nu ){]}^{\frac{q}{2}}\}}^{\frac{1}{q}}$$

Where $q$ is an index which can take all possible values except zero because in that case the factor 1/$q$ blows up. *F*_*q*_ cannot be obtained by the normal averaging procedure; instead a logarithmic averaging procedure is applied

4 $${F}_{0}(s)\equiv \exp \{\frac{1}{4{N}_{s}}\sum _{\nu =1}^{2{N}_{s}}\mathrm{ln}\,[{F}^{2}(s,\nu )]\} \sim {s}^{h(0)}$$

**Step 5:** The procedure is repeated by varying the value of *s*.*F*_*q*_(*s*) increases with increase in value of $s$. If the series is long range power correlated, then *F*_*q*_(*s*) will show power law behaviour

$${F}_{q}(s)\propto {s}^{h(q)}$$

If such a scaling exists $\mathrm{ln}\,{F}_{q}(s)$ will depend linearly on ln *s* with *h*(*q*) as the slope. In general the exponent *h*(*q*) depends on $q$. For stationary time series *h*(2) is identical with the Hurst exponent H. *h*(*q*) is said to be the generalized Hurst exponent. A monofractal time series is characterized by unique *h*(*q*) for all values of $q$.

The generalized Hurst exponent *h*(*q*) of MF-DFA is related to the classical scaling exponent $\tau (q)$ by the relation

5 $$\tau (q)=qh(q)-1$$

A monofractal series with long range correlation is characterized by linearly dependent $q$ order exponent $\tau (q)$ with a single Hurst exponent H. Multifractal signal have multiple Hurst exponent and $\tau (q)$ depends nonlinearly on $q$ ^40^.

The singularity spectrum $f(\alpha )$ is related to $\tau (q)$ by a Legendre transformation. It is related to h(q) by

6 $$\alpha =h(q)+qh^{\prime} (q)$$

7 $$f(\alpha )=q[\alpha -h(q)]+1$$

where *α* is the singularity strength and $f(\alpha )$ specifies the dimension of subset series that is characterized by *α*. Unique Hölder exponent denotes monofractality, while in the multifractal case, the different parts of the structure are characterized by different values of *α*, leading to the existence of the spectrum $f(\alpha )$. The multifractal spectrum is capable of providing information about relative importance of various fractal exponents in the series e.g. the width of the spectrum denotes range of exponents. A quantitative characterization of the spectra may be obtained by least square fitting it to a quadratic function^41^ around the position of maximum *α*_0_,

8 $$f(\alpha )=A{(\alpha -{\alpha }_{0})}^{2}+B(\alpha -{\alpha }_{0})+C$$

where C is an additive constant ${\rm{C}}=f({\alpha }_{0})=1$. B indicates the asymmetry of the spectrum. It is zero for a symmetric spectrum. A right skewed spectrum with B > 0 indicates dominance of high fractal exponents and hence presence of fine structure while B < 0 suggests smooth structure. The width of the spectrum can be obtained by extrapolating the fitted curve to zero. Width *W* is defined as

9 $$W={\alpha }_{2}-{\alpha }_{1}$$

with $f({\alpha }_{1})=f({\alpha }_{2})=0$. It has been proposed by some groups^42^ that the width of the multifractal spectra is a measure of degree of multifractality. For a monofractal series, *h*(*q*) is independent of $q$. Hence from relation (6) and (7) it follows that we have a unique value of α for all values of q and $f(\alpha )=1$. Hence the spectrum collapses to a single point so that the width of the spectrum will be zero for a monofractal series. The more the width, the more multifractal is the spectrum.

The above definition of the width of the spectra is equivalent to expressing degree of multifractality by (*h*_*max*_−*h*_*min*_)^43^. Though other methods of degree of multifractality have been proposed^44^, but the current method discussed above has the advantage that it takes into account negative moment orders q as well, thus information hidden in large as well as small fluctuations can be captured^43^.

The origin of multifractality can be determined in the following way:

There are two basic sources of multifractality, (i) Multifractality due to broad probability density function (ii) Multifractality due to different long-range correlations of the small and large fluctuations.

The origin of the multifractality can be ascertained by analyzing the corresponding randomly shuffled series. In the shuffling procedure, the values are put into random order and hence all correlations are destroyed. Hence, if the multifractality is due to long-range correlations, then the shuffled series exhibits a non-fractal scaling. On the other hand, if the original *h*(*q*) dependence does not change, i.e. $h(q)={h}_{shuffled}(q)$, then the multifractality is due to the broad probability density, which is not affected in the shuffling procedure. If both kinds of multifractality are present in a given series, the shuffled series will show weaker multifractality than the original series.

The autocorrelation exponent $\gamma$ can be estimated from the relation given below:^17, 45^

10 $$\gamma =2-2h(q=2)$$

For uncorrelated or short-range correlated data, *h*(2) is expected to have a value 0.5 while a value greater than 0.5 is expected for long-range correlations. Therefore for uncorrelated data, $\gamma$ has a value 1 and the lower the value the more correlated is the data.

## Results and Discussion

**Figure 3:**
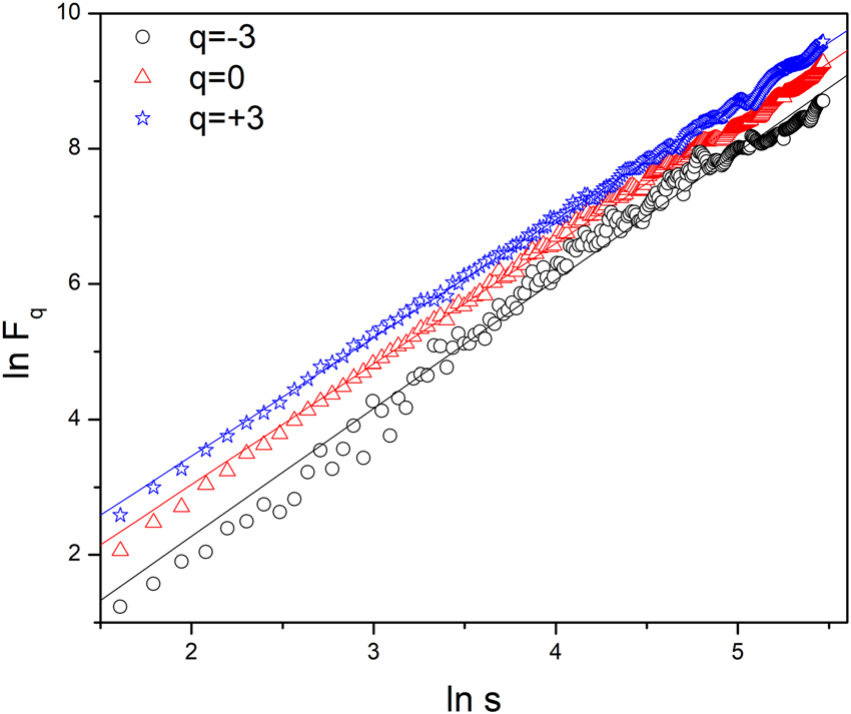
ln Fq vs. lns plots illustrating scaling relationship for a particular dataset. Linear Detrending was used to estimate the Fluctuation function Fq(s). s was varied from 5 to N/4, where N is the length of the data. q was chosen from −3 to + 3 in steps of 0.1. For clarity of representation plots for only q = −3, 0, + 3 is shown.

**Figure 4:**
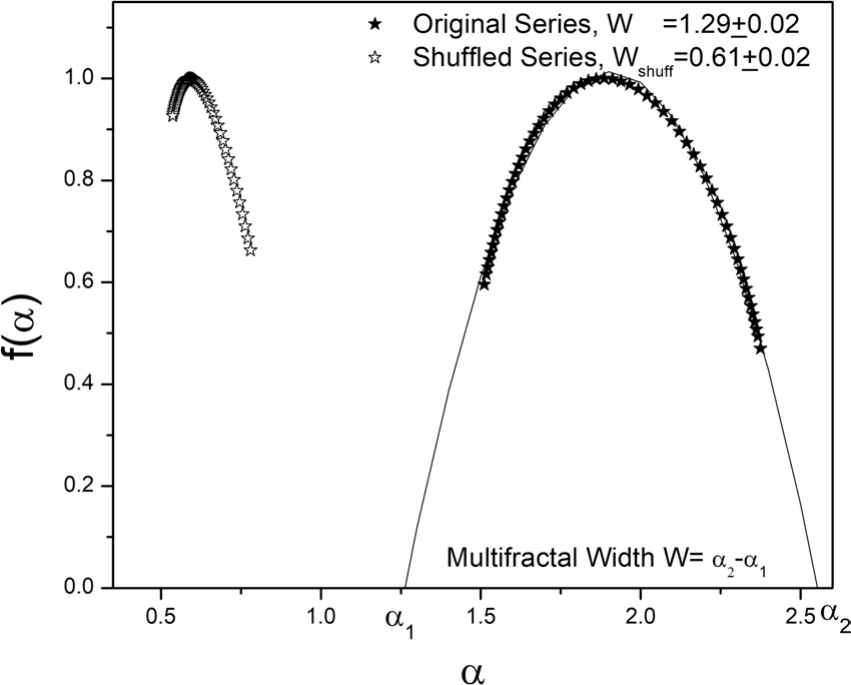
Multifractality spectrum f(α) vs. α for original and shuffled series for a particular set. The width of the spectrum can be obtained by extrapolating the fitted curve to zero. Width *W* is defined as *W* = *α*_2_ − *α*_1_ with *f*(*α*_1_) = *f*(*α*_2_) = 0. The shuffled series exhibits weaker multifractality. The width of the spectrum diminishes considerably for the shuffled series indicating that long-range correlations are primarily responsible for origin of multifractality of the elevation profile.

The glacial and fluvial landscapes were analyzed using the MFDFA methodology. The fluctuation function was estimated for each set according to eqn 3 and 4. The scaling property of the fluctuation function is depicted in Fig. 3. The scale *s* was varied to the maximum value N/4 (as allowed in MFDFA methodology). Linear relation was observed for all length scales and no cross-over was observed. The multifractal spectrum was obtained from 6 and 7. Multifractal width was estimated for each dataset by fitting the spectrum $f(\alpha )$ vs. *α* (refer to Fig. 4) to the eqn 8. The origin for multifractality was ascertained by analyzing the shuffled series. Figure 4 shows the multifractal spectrum $f(\alpha )$ vs. *α* for original series and corresponding randomly shuffled series for a particular dataset. The multifractal width for original series (1.29 $\pm$ 0.02) is largely reduced for the shuffled series (0.61 $\pm$ 0.02). Thus we can conclude that long range correlations are primarily responsible for the origin of multifractality in the landscapes under consideration.

**Figure 5:**
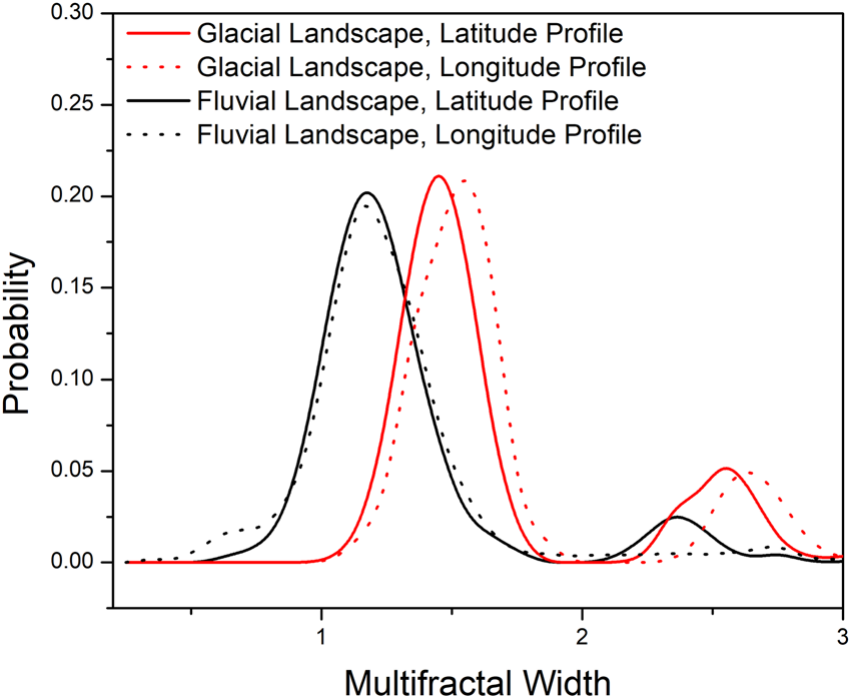
Distribution of Multifractal Width W.

The distribution of multifractal widths is shown in Fig. 5. Table 1 depicts the mean multifractal width and variance for latitude and longitude profiles for glacial and fluvial landscapes. A nearly zero p-value was observed which signifies a 100% confidence level that the mean values obtained are significantly different for glacial and fluvial landscapes for both latitude and longitude profiles. A higher value of W for glacial landscapes compared to the fluvial one suggests that the glacial landscapes exhibits more complex structures than fluvial landscapes.

**Table 1:** Mean, Variance and p-values of Multifractal Width W, Correlation coefficient *γ*, Asymmetry Index B and Dominant Holder Exponent *α*_0_ for Glacial and Fluvial Landscape.

Profile	Parameter	Landscape	Mean	Variance	p-value
Latitude	Multifractal Width W	Glacial	1.76	0.4	$\approx$0
		Fluvial	1.33	0.2	
Longitude		Glacial	1.78	0.4	$\approx$0
		Fluvial	1.29	0.2	
Latitude	Correlation Coefficient *γ*	Glacial	0.609	0.007	$\approx$0
		Fluvial	0.365	0.006	
Longitude		Glacial	0.735	0.01	$\approx$0
		Fluvial	0.583	0.02	
Latitude	Asymmetry Index B	Glacial	0.23	0.02	0.60
		Fluvial	0.22	0.03	
Longitude		Glacial	0.23	0.02	$\approx$0
		Fluvial	0.15	0.04	
Latitude	Dominant Holder Exponent *α*_0_	Glacial	1.886	0.002	1.1 × 10^–15^
		Fluvial	1.902	0.001	
Longitude		Glacial	1.836	0.004	$\approx$0
		Fluvial	1.783	0.003	

ANOVA parameter p-value represents significance of differences between group means of glacial and fluvial landscapes.

Glacial landscapes shows two peaks in the multifractal width distribution, a prominent peak at around 1.5 and a lesser prominent peak at around 2.6 with a dip in between. By applying ANOVA to the multifractal width values for latitude and longitude profiles of elevation of glacial landscapes, we have obtained a p-value 0.4 for the glacial landscapes. Thus the distribution of multifractal width for glacial landscapes is nearly same for the latitude and the longitude profiles.

The same is not true for fluvial landscapes. Figure 5 shows that though the distribution for W peaks at nearly same value 1.15 for the latitude and longitude profiles, but the latitude profile exhibits a lesser prominent peak at 2.35 which is absent for the longitude profiles. Applying ANOVA to the W values of latitude and longitude profiles of elevation of fluvial landscapes a p-value as low as 0.07 was obtained which signifies that the means for latitude and longitude profiles for fluvial landscapes are significantly different. Thus the Himalayan fluvial landscapes reveal spatial anisotropy along the two directions. The same has also been observed in other fluvial landscapes^46^.

It was also observed that clustering of regions having high multifractal width is denser along the latitude, or in other words the longitude profiles at given latitude with high degree of multifractality are clustered densely. Clustering is also observed along longitude but it is not so dense. However no clustering of high W regions has been observed for fluvial landscapes. It is also observed that the elevation profiles of high W regions are morphologically identical with U shaped glacial valleys.

**Figure 6:**
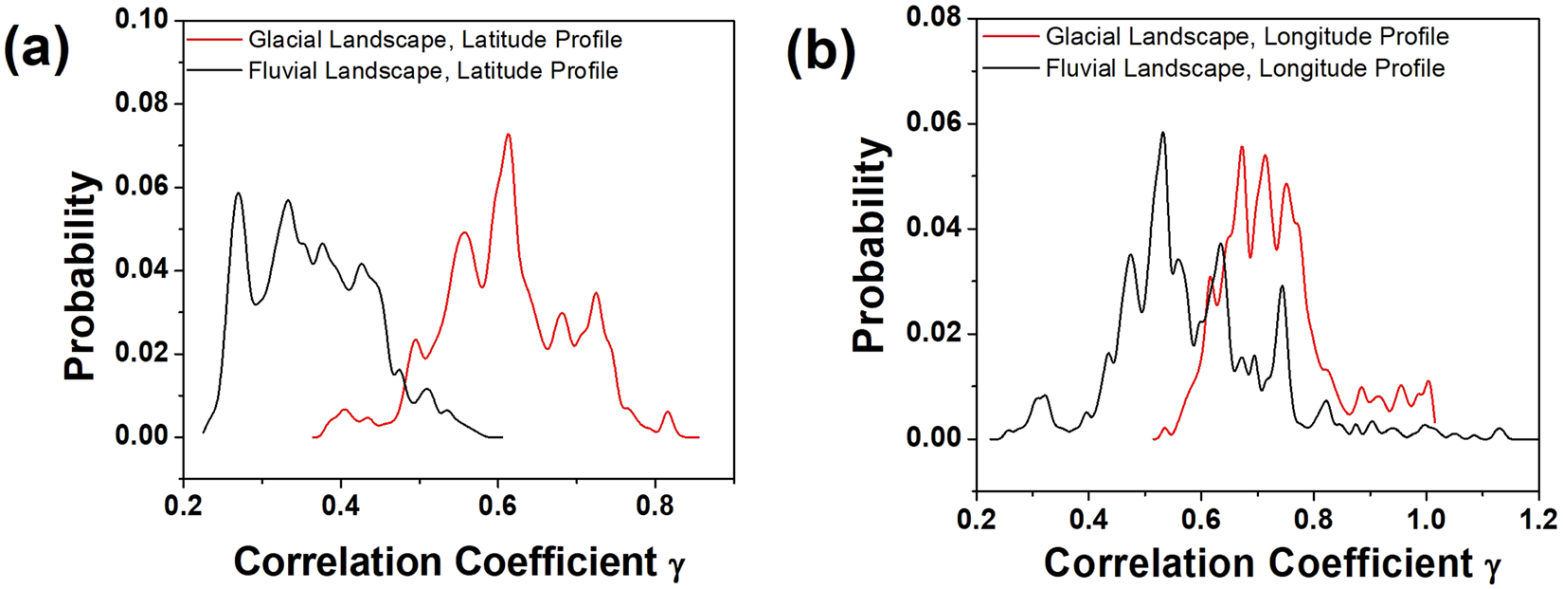
Distribution of Correlation coefficient *γ* for (**a**) Latitude Profile and (**b**) Longitude Profile. For clarity of presentation the distribution of *γ* for latitude and longitude profiles have been shown separately.

We have also determined the variation of other fractal parameters such as the correlation strength $\gamma$ defined by eqn 10. Figure 6 shows distribution of *γ*. The lower the value of *γ*, more correlated is the data. The fluvial landscapes are found to be more correlated with respect to the glacial ones (see Table 1). It is also interesting to observe that the latitude profiles are more correlated both in glacial and fluvial landscapes compared to the longitude profile. A possible reason might be the fact that both the glacial and fluvial valleys are oriented along the N-S direction as shown in Fig. 1(b) and (c), hence the latitude profiles seems to be more correlated compared to the longitude profiles.

**Figure 7:**
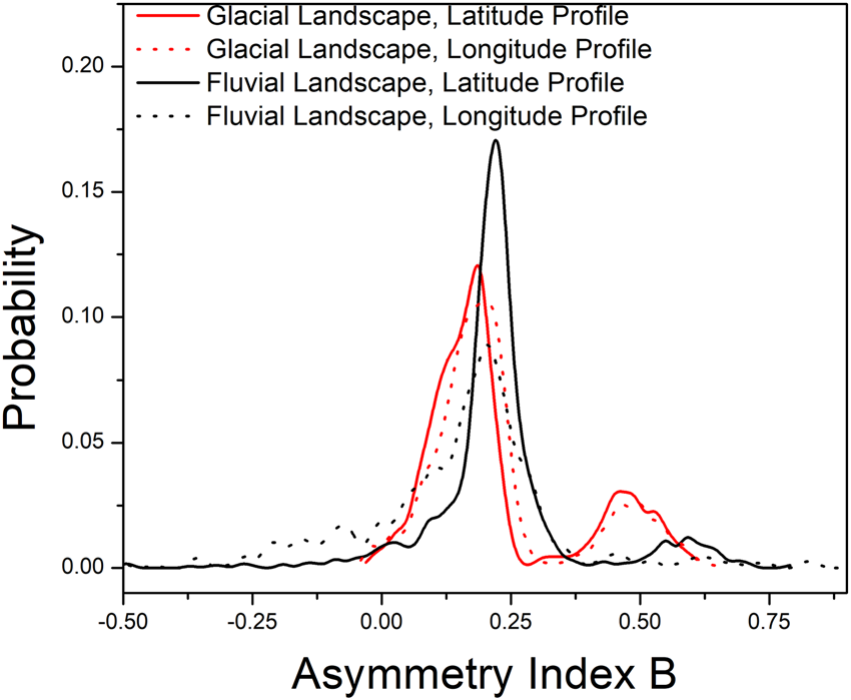
Distribution of Asymmetry Index B.

The variation of asymmetry index B as obtained from fitting the multifractal spectrum to eqn 8 was also studied and is depicted in Fig. 7. B > 0 suggests fine structure while B < 0 suggests presence of smooth structure. While B was found to be identical for both directions in glacial landscapes, anisotropy was observed in case of fluvial landscapes. A higher value of B suggests more roughness in the terrain while a comparatively lower value suggests that the terrain is smoother. Gagnon *et al*.^19^ have shown that multifractality can reveal amalgamation of rough and smooth terrains in different proportions in earth topography. Though the latitude profile of fluvial valleys exhibits complex structure similar to the glacial ones, the longitude profiles exhibit a smoother structure. Thus along with the multifractal width the asymmetry index also reflects the anisotropic structure of fluvial valleys.

**Figure 8:**
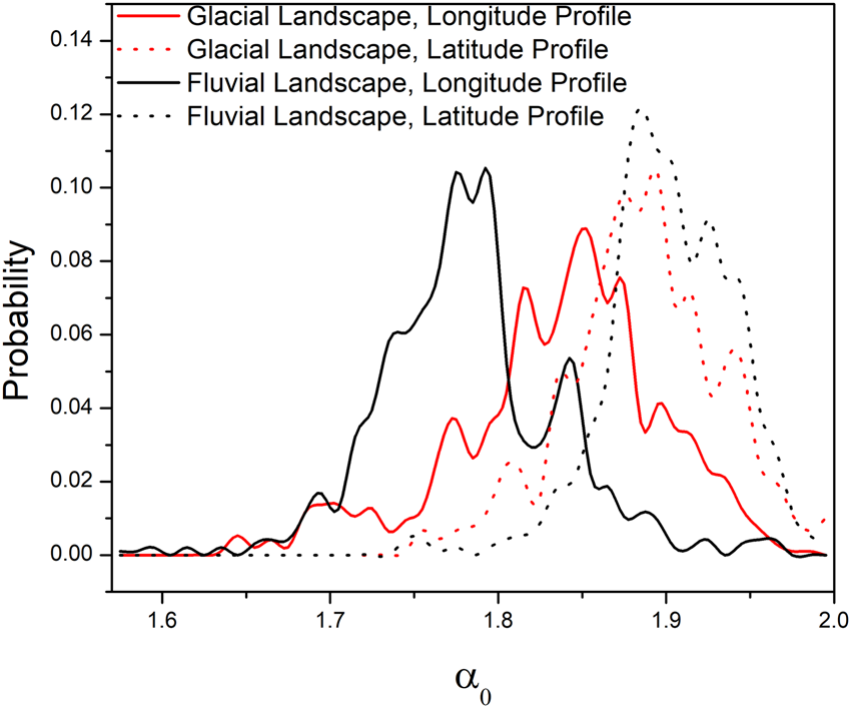
Distribution of Dominant Holder Exponent *α*_0_.

The distribution of dominant Holder exponent *α*_0_ was also studied and depicted in Fig. 8. The distributions of *α*_0_ for glacial and fluvial landscapes are quite similar for the latitude profiles. The value of *α*_0_ (see Table 1) was found to significantly different for glacial and fluvial landscapes, but the change is not parallel in both directions. Hence it is not possible to consistently correlate the values of *α*_0_ with morphological characteristics as successfully done in case of other fractal parameters.

## Conclusions

The study of multifractal properties of glacial and fluvial landscapes has revealed the following interesting features:

(i) Both glacial and fluvial landscapes depict complex multifractal structure, which may be attributed to long range correlations.
(ii) The glacial landscapes are more complex in nature as evident from multifractal width and asymmetry parameter. The variation of elevation profile along the latitude and longitude are approximately isotropic.
(iii) Fluvial landscape shows less complex structure and the valleys seem to be anisotropic along the two directions. The mean multifractal widths as well as asymmetry parameters are statistically different for the latitude profile and longitude profile.

The above study has revealed some interesting conclusions. It may be also tested for other Himalayan Glaciers and Glacial and Fluvial data over the world to check whether the properties are universally applicable to all glaciers. However, this study has certain limitations. We have performed one dimensional MFDFA but it is a well known fact that the fluctuation gets reduced when we look into them in lower dimensions. Therefore a higher dimensional analysis like 2DMFDFA, 2DMFDMA, Wavelet analysis would be more appropriate^47–49^. We have also observed that the glacial valleys are more or less isotropic in nature and fluvial valleys reflect anisotropy. However, the present analysis is unable to throw light as to what are the underlying reasons behind these observations. A very recent work on two dimensional wavelet analysis of fluvial valleys has been reported in this respect^46^. Nevertheless this paper seeks to present some new and interesting data on Himalayan glaciers which has not been reported in past.
